# Barriers and facilitators to implementing priority inpatient initiatives in the safety net setting

**DOI:** 10.1186/s43058-020-00024-6

**Published:** 2020-03-11

**Authors:** Erika L. Crable, Dea Biancarelli, Allan J. Walkey, Mari-Lynn Drainoni

**Affiliations:** 1 Evans Center for Implementation and Improvement Sciences, Boston University School of Medicine, 801 Massachusetts Avenue, Crosstown 2030, Boston, 02118 MA USA; 2 Department of Health Law, Policy & Management, Boston University School of Public Health, Boston, MA USA; 3 The Pulmonary Center, Department of Medicine, Boston University School of Medicine, Boston, MA USA; 4 Section of Infectious Diseases, Department of Medicine, Boston University School of Medicine, Boston, MA USA; 5 Center for Healthcare Organization and Implementation Research, Edith Nourse Rogers Memorial VA Hospital, Bedford, MA USA

**Keywords:** Safety net, Hospital, Implementation, Improvement, Qualitative methods, Consolidated Framework for Implementation Research

## Abstract

**Background:**

Safety net hospitals, which serve vulnerable and underserved populations and often operate on smaller budgets than non-safety net hospitals, may experience unique implementation challenges. We sought to describe common barriers and facilitators that affect the implementation of improvement initiatives in a safety net hospital, and identify potentially transferable lessons to enhance implementation efforts in similar settings.

**Methods:**

We interviewed leaders within five inpatient departments and asked them to identify the priority inpatient improvement initiative from the last year. We then conducted individual, semi-structured interviews with 25 stakeholders across the five settings. Interviewees included individuals serving in implementation oversight, champion, and frontline implementer roles. The Consolidated Framework for Implementation Research informed the discussion guide and a priori codes for directed content analysis.

**Results:**

Despite pursuing diverse initiatives in different clinical departments, safety net hospital improvement stakeholders described common barriers and facilitators related to inner and outer setting dynamics, characteristics of individuals involved, and implementation processes. Implementation barriers included (1) limited staffing resources, (2) organizational recognition without financial investment, and (3) the use of implementation strategies that did not adequately address patients’ biopsychosocial complexities. Facilitators included (1) implementation approaches that combined passive and active communication styles, (2) knowledge of patient needs and competitive pressure to perform well against non-SNHs, (3) stakeholders’ personal commitment to reduce health inequities, and (4) the use of multidisciplinary task forces to drive implementation activities.

**Conclusion:**

Inner and outer setting dynamics, individual’s characteristics, and process factors served as implementation barriers and facilitators within the safety net. Future work should seek to leverage findings from this study toward efforts to enact positive change within safety net hospitals.

Contributions to the literature

- This research identifies and describes implementation barriers and facilitations that are specific to safety net settings, as experienced by implementation champions, frontline implementers, and individuals in implementation oversight/leadership roles.
- Application of the Consolidated Framework for Implementation Research at a macro-level provides a common terminology to describe inner and outer setting, process, and individual characteristics that influence the uptake of new initiatives.
- Better understanding barriers and facilitators to implementing priority initiatives in safety net settings can inform future optimization of implementation strategies that are tailored to the safety net setting, patient, and provider needs.

## Background

Hospitals face persistent pressure to improve the quality and safety of patient care. Nearly 20 years have passed since the Institute of Medicine’s *To Err is Human* [1] and *Crossing the Quality Chasm* [2] reports highlighted the prevalence of preventable medical errors and quality challenges across the US healthcare system. Yet many problems described persist due to the real-world difficulty of translating evidence-based practices into routine clinical workflow [3]. Hospitals also face financial pressure to improve, as pay-for-performance initiatives tie incentive payments to a variety of care and process outcomes [4]. In response to financial pressure to improve performance, many hospitals have scaled up in-house quality improvement programs to tackle unremitting issues, for example, low rates of hand-hygiene compliance, underuse of evidence-based practice protocols, and delayed discharge processes [5–7].

Prior research on facilitators of hospital improvement efforts have highlighted diverse resource-intensive (e.g., monetary, time) interventions and strategies to promote systems change or teamwork cultures that support change efforts [8, 9]. However, little is known about interventions that drive improvements in low-resource settings, such as safety net hospitals (SNH), which may be less able to implement resource-intensive initiatives [10].

SNHs deliver health services primarily to Medicaid beneficiaries, the uninsured, under-insured, and other vulnerable groups, regardless of their ability to pay [11]. SNHs often operate with thin budgets and rely on Disproportionate Share Hospital (DSH) payments to help offset the costs of uncompensated care [12]. Research suggests that settings with limited financial resources are associated with reduced innovation in healthcare [13, 14]. SNHs may have even less tolerance than other hospitals for financial risks associated with improvement initiatives, as planned reductions in DSH payments further constrain their operating budgets [15].

It is uncertain whether SNHs experience the same barriers to improvement as non-SNHs [10]. Prior research in non-SNHs has identified staff reluctance to take on implementation responsibilities that stretch beyond current work roles as one barrier to adoption of new practice changes [16, 17]. However, staff hesitancy may be less of an issue among safety net providers who choose to work in settings with fewer resources and expect to serve in boundary-spanning roles [18, 19]. While research suggests insufficient resources delay hospital implementation efforts [20], it is unclear whether SNH constraints slow or prohibit innovation. The complex patient population treated in SNHs may also create barriers to improvement. For example, efforts focused on improvement in one department may be insufficient to enhance outcomes for patients with co-morbidities who require care across departments. Language and cultural discordance between patients and providers can also hinder efforts to implement patient-centered care initiatives [21, 22]. SNH-specific implementation research is needed to identify key factors that influence the uptake of evidence-based innovations and determine how they differ from drivers of change in non-SNHs.

This study aimed to identify barriers and facilitators that affect the implementation of priority initiatives in the inpatient units of an urban SNH and offer recommendations to improve SNH implementation efforts. By interviewing diverse stakeholders across five different inpatient units, we aimed to describe potentially transferable lessons about factors that hinder or promote implementation in a SNH serving a diverse patient population.

## Methods

### Theory

The Consolidated Framework for Implementation Research (CFIR) guided the research. CFIR is a comprehensive framework that synthesizes core constructs from 29 prominent organizational and implementation science theories, with uniform language to promote generalizability across disciplines [23]. CFIR describes five domains theorized to influence implementation success: [1] intervention characteristics, [2] inner setting, [3] outer setting, [4] characteristics of the individuals involved, and [5] the process of implementation. We chose CFIR because it is not specific to any intervention type (e.g., increasing protocol use) and allowed us to investigate common implementation barriers and facilitators across diverse interventions.

Our application of CFIR is novel. Although CFIR has been widely used in implementation science research to describe how individual interventions are promoted and adopted [24], this is one of the first studies to use CFIR as a tool for collectively analyzing the implementation experiences, barriers, and facilitators of multiple, programmatically distinct initiatives. Applying CFIR in this way leverages the framework’s ability to synthesize core factors influencing implementation processes and reveal meaningful themes across diverse initiatives whose only initial commonality was that implementation occurred in a SNH.

### Study setting and sample

We conducted qualitative interviews to assess implementation efforts underway in five inpatient units in an urban SNH. The 514-bed SNH provides a full spectrum of pediatric and adult care outpatient and inpatient services, with over 1.1 million patient visits and 25,000 admissions annually. Over 70% of patients come from underserved populations including individuals who are low-income, and Medicaid and/or Medicare beneficiaries. Almost one third of the SNH patients do not speak English as a primary language. Researchers (ELC, MD) initially emailed chairs of five large departments, asking them to identify their priority inpatient implementation initiatives during the previous 12 months. All reported ongoing implementation efforts and identified key stakeholders involved in those initiatives. We requested individual, confidential interviews with the department chairs and the other identified key stakeholders via email, and used snowball sampling techniques throughout the study to identify additional key informants, including physicians, nurses, and other frontline staff involved in each priority initiative.

### Data collection

The publicly available CFIR Interview Guide Tool [25] informed our initial interview guide. We tailored the guide to promote a semi-structured interview style that included questions about implementing initiatives in a SNH (Supplemental file 1). Interviews began by asking participants to describe the most important initiative that their department tried to implement or maintain in the last year. Subsequent questions focused on the priority initiative described by the key informant, even if that initiative differed from the effort originally identified by the department chair. Additional questions addressed how the initiative was prioritized, whom the intervention targeted, individual’s specific role(s) in implementing or maintaining initiatives, personal beliefs about the initiative being implemented, implementation processes, internal or external environmental factors that influenced implementation, and barriers or facilitators to implementation.

Two trained qualitative researchers (ELC, DB) conducted individual, semi-structured interviews with key informants between January 2018 and May 2018. Interviews lasted between 30 and 45 min, were audio-recorded, and professionally transcribed. Data collection continued until the research team (ELC, DB, AJW, MD) agreed that thematic saturation was met such that participant interviews revealed common experiences across CFIR constructs, and new insights were unlikely to be obtained through additional interviews [26, 27].

### Data analysis

We conducted a directed content analysis of interview transcripts to identify themes describing barriers and facilitators to implementation as they relate to core CFIR constructs. The directed content approach allowed us to extend the application of CFIR’s core constructs across multiple inpatient settings, initiatives, and implementation experiences [28]. Core CFIR constructs were identified as a priori codes for an initial codebook. Prior to coding, the research team reviewed and discussed CFIR coding definitions presented by Damschroder et al. (2009) to come to a collective understanding of the a priori codes. Due to the expansiveness of CFIR constructs, two coders (ELC, DB) independently double-coded all transcripts. Coders met regularly to review coding consistency and discuss problematic constructs, such as distinguishing “intervention complexity” and “intervention compatibility,” and achieve team consensus. A senior qualitative researcher experienced in applying CFIR constructs (MD) resolved disagreements in coding decisions. We maintained a log of all final coding decisions and construct clarifications. Two coders (ELC, DB) reviewed data coded to each construct and identified preliminary themes related to each CFIR construct and the larger CFIR domains. The larger research team (ELC, DB, AJW, MD) discussed preliminary themes to reach consensus on final deductive themes. All coding and analysis were conducted in NVivo [29].

### Ethics review and reporting standards

This study was determined to be exempt by the Boston University Medical Campus Institutional Review Board (H-36161). The Standards for Reporting Qualitative Research checklist guided our reporting of the qualitative methods and results (Supplemental file 2) [30].

## Results

### Participant characteristics

Thematic saturation was achieved after conducting interviews with 25 stakeholders involved in various aspects of implementation (Table 1). We identified three types of discrete roles: implementation oversight, implementation champion, and frontline implementers. Individuals in implementation oversight roles (*n* = 5) provided formal approval for initiatives, but were not involved in day-to-day implementation activities. They served in hospital leadership positions, such as department chairs or chiefs, or patient safety and quality leaders. Implementation champions (*n* = 8) directed implementation efforts and were critical to engaging others in initiatives. Champions were primarily physicians (*n* = 4) or nurses (*n* = 3). Frontline implementers (*n* = 12) included individuals whose daily workflow activities were directly impacted by department initiatives; most were physicians (*n* = 7).

**Table 1:** Stakeholders interviewed by role in implementation efforts

*Stakeholder type*	Implementation oversight (*n* = 5)	Implementation champion (*n* = 8)	Frontline implementer (*n* = 12)
Hospital leadership	5	1	1
Physicians	0	4	7
Nurses	0	3	4

### Description of the inpatient initiatives

Participants identified eight diverse priority initiatives across the five inpatient settings. To preserve participant confidentiality and because this study aimed to compare barriers and facilitators experienced during various SNH implementation activities, initiatives are described in terms of their improvement goal foci. Two initiatives aimed to improve communication and workflow processes to achieve efficiencies in service delivery. Six initiatives aimed to reduce practice variation and improve clinical outcomes by promoting the uptake of evidence-based practices outlined in guidelines and protocols.

### Themes

Directed content analysis revealed seven themes across four CFIR domains: inner setting, outer setting, characteristics of individuals involved, and implementation process. Of the seven themes that emerged across all initiatives, three represented barriers and four represented facilitators to implementation. Descriptions of the themes and their impact on implementation efforts are described below and summarized in Table 2.

**Table 2:** Summary of themes by Consolidated Framework for Implementation Research (CFIR) domain and constructs

CFIR domain	Key constructs	Themes as barriers or facilitators to implementation efforts
Inner setting	• Available resources • Organizational incentives and rewards • Access to knowledge and information	Barrier: Limited resources delayed implementation efforts and uptake of innovations
		Barrier: Organizational recognition is critical to sustaining initiatives, but is not sufficient without financial investment
		Facilitator: Implementation approaches that combined passive and active communication styles promoted initiative fidelity and sustainability
Outer setting	• Needs and resources of those served by the organization • Peer pressure • External policy & incentives	Facilitator: Knowledge of patient needs and competitive pressure spurred innovation
		Barrier: Implementation strategies that did not adequately address patients’ biopsychosocial complexities delayed initiative progress
Characteristics of individuals involved	• Individual identification with organization • Other personal attributes	Facilitator: Individuals’ personal commitment to reducing health inequities in the safety net population motivated initial participation and ongoing support for new initiatives
Implementation Process	• Planning • Engaging	Facilitator: Multidisciplinary task forces conducted successful implementation efforts

### Domain 1: Inner setting

Within the inner setting, we identified two barriers and one facilitator to implementing priority initiatives.

#### Barrier: Limited resources delayed implementation efforts and uptake of innovations

Within the SNH’s inner setting, limited *available resources* organizationally dedicated to implement and support ongoing initiatives hindered the uptake of innovations. Stakeholders across implementation roles identified limited access to resources such as protected time for projects, staff to support data collection or analytics, and research training as barriers to initiating and sustaining new initiatives. Champions were frustrated by the lack of protected time to work on department-approved projects. Many described working on improvement initiatives during their personal time. Some frontline implementers said they were not able to participate in meetings related to implementation planning or progress updates because they were unable to secure time away from clinical duties.

*If you have a project or you want to do something, you have to figure out how to get the time off, or do it on your day off. You know, and that’s hard. - Implementation Champion 16*

None of the priority initiatives had dedicated project managers or staff to support data collection and analysis. Champions often also served as project managers, data collectors, and analysts, but described feeling inadequately trained for these roles.

*I think that we would have been going much faster if we had existing staff that we could tap into for data management and data collection, in conjunction with project management support. Those are the most important things. – Implementation Champion 01*

Without the ability to achieve timely data reporting, implementation champions struggled to assess progress toward project benchmarks. Champions felt overcommitted and underprepared, and described being in a constant state of reaction to, rather than prevention of, implementation challenges.

*The majority of the people who work here already work very hard, and so taking on one more thing is not a small ask. So being able to at least say ‘I understand what it is that’s needed of me and being asked of me’… It would have meant I was in a more proactive stance I think in terms of recruiting the right people. Um, and less reactive. – Implementation Champion 04*

#### Barrier: Organizational recognition is critical to sustaining initiatives, but is not sufficient without financial investment

Several stakeholders described receiving *organizational incentives* in the form of congratulatory emails and hospital awards for their efforts in the design and implementation of improvement initiatives. Participants viewed organizational recognition as an important motivator for increasing participation in initiatives.

*I think it always helps to have a reward and feel like you’ve accomplished something. – Frontline Implementer 17*

However, stakeholders juxtaposed receipt of organizational rewards with what they described as an insufficient financial investment from the hospital in successful initiatives. Participants in all roles said it was acceptable to use department resources to pilot new initiatives, but felt the hospital should commit to funding projects for long-term sustainability after they were proven successful.

*I just can’t do them all myself. It would be nice if [SNH leader] or someone from [the SNH leadership group] would come in and say, 'Alright, this is, um, this needs some more direction from the hospital quality group'' and then they helped us. – Implementation Oversight 08*

Participants who were engaged in projects that directly helped the hospital achieve quality benchmarks wanted the hospital to use initiative-related savings to sustain and scale-up efforts. Without greater financial support, many implementation overseers and champions questioned the sustainability of their initiatives.

*We have a successful intervention, and we’re not allowed to scale it up because of the usual problems in [the SNH] with lack of space and lack of staff and lack of physician and nursing time and the like… At some point, you know, if you do quality improvement that’s successful and you integrate it in what you’re doing, it sometimes takes – I think this is part of the maintenance phase – it takes resources to keep it going once that initial enthusiasm and interest... But no one says, ‘Oh, you guys have-have-have decreased the, um, the cost…Now we’ll, you know, provide you with some other services to make up for that’. – Implementation Oversight 10*

#### Facilitator: Implementation approaches that combined passive and active communication styles promoted initiative fidelity and sustainability

When *access to knowledge and information* about initiatives was communicated via diverse active and passive approaches, initiatives thrived. However, initiatives that relied solely on passive strategies, such as emails or posters, to educate staff about new workflow or treatment processes experienced poor and inconsistent uptake. Although several stakeholders identified problems with disseminating new practices via “word of mouth,” informal methods of communicating new knowledge about initiatives were common and not considered useful.

*I think the concept was each discipline was gonna cascade information to their own group… But if you have heard information, you know, about something you haven't done before six months ago and, you know, it’s not something you do every day, six months later you gonna remember that? No… So, it’s frustrating when you just, you feel like it's Groundhog Day sometimes. – Implementation Champion 19*

Implementation teams that used a combination of passive and active communication approaches experienced greater information sharing and uptake. Effective passive strategies included written information provided via the hospital’s online learning portal, didactic presentations about planned changes, and emails and stationary binders containing resources related to the initiative. These passive strategies were only successful when combined with active training sessions and physical demonstrations of new procedures. Training frontline implementers in small groups or one-on-one promoted initial uptake and sustained the use of new processes, because small discussions enabled champions to describe the rationale and evidence for practice changes. Physical demonstration of new tasks was also critical to promoting fidelity to complex initiatives.

*Education was done via Health Stream, PowerPoint slides, things like that. Any place that we can connect is where we’ll go. – Implementation Champion 12*

*It was almost like a one-to-one, like, you know, we are going to each one of them, we had a little folder where we had those guidelines for them to see and then all the evidence to back up, so we, like, talked to them one to one, went through the guidelines. Sometimes they could be group discussions but we tried to do it one-to-one. – Frontline Implementer 07*

### Domain 2: Outer setting

Stakeholders’ *knowledge of the needs and resources of individuals served* by the SNH and *peer pressure* to compete with other hospitals around *external policies and incentives* like publicly reported quality benchmarks revealed two outer setting themes.

#### *Facilitator:* Knowledge of patient needs and competitive pressure spurred innovation

Participants across implementation oversight, champion, and frontline implementer roles were highly aware of safety net population concerns, such as comorbidities, homelessness, economic constraints, and other social factors associated with poor health outcomes. Participants frequently described treating a “patient population [that] is incredibly high risk” (Frontline Implementer 20) and perceived as sicker than the patients treated at other area hospitals. Individuals in implementation oversight roles were eager to suggest or approve of new initiatives that targeted improvements in publicly reported patient outcomes. Both implementation overseers and champions wanted to show that despite treating a sicker population, they could boost the SNH’s public rankings against non-SNHs. Competitive pressure to improve quality benchmarks was a key driver for identifying and rallying support for new initiatives.

*It was something, you know, that we had to find some solution for, ‘cause it-it wasn’t acceptable. Everybody else was having such a lower rate. – Frontline Implementer 16*

#### Barrier: Implementation strategies that did not adequately address patients’ biopsychosocial complexities delayed initiative progress

Despite widespread knowledge of patient needs, frontline implementers reported that initiatives frequently did not adequately consider implementation challenges posed by patients’ complex care and socioeconomic needs.

*It’s always a lot harder. Like, you can say this is what we’re gonna do, and then there’s always seven thousand things that happen in the interim that make it difficult. There’s the ideal situation and then you figure out that, like, 10 to 20 percent of our patients will fit into that ideal situation.- Frontline Implementer 21*

Initiatives focused on achieving efficiencies in clinical workflow and care delivery were thwarted by communication delays between multiple specialty care teams that were needed to treat patients with complex comorbidities. Frontline implementers and champions also described slow uptake of initiatives due to provider-patient language discordance. Implementation progress temporarily slowed when patient-facing educational materials needed to be revised or translated. Initiatives that included length of stay as a measure of success experienced little progress due to the high prevalence of patients who were medically ready for discharge but who lacked housing or transportation.

*You walk into the room and they’re like ‘I have nowhere to go, can I at least stay through lunch? I like want to have another hot meal.’ You-you know? Your heart’s going to-going to break if you’re like ‘nope’. So that person I'm not going to force out the door at 10 AM. I'm going to like let them stay to eat their lunch if I feel like they really should. I feel like it’s tugging at my heart strings, which isn’t necessarily the best thing for the hospital… but feels like the right thing to do in the moment as a person and a human. – Frontline Implementer 24*

### Domain 3: Characteristics of individuals involved

*Individuals’ identification with the SNH* and their own *personal attributes* converged to reveal a critical facilitator of priority initiatives in the SNH.

#### Facilitator: Individuals’ personal commitment to reducing health inequities in the safety net population motivated initial participation and ongoing support for new initiative

Synergy between individuals’ personal creed to reduce health disparities and the hospital’s mission to provide quality care to the safety net population was a predominant facilitator of implementation success. Stakeholders in implementation oversight, champion, and frontline implementer roles described themselves and colleagues as having a shared purpose of caring for the safety net population. Individuals’ cited their personal motivation to reduce health disparities as a rationale for choosing to work at the SNH and for regularly engaging in implementation efforts to improve care. This synergy promoted stakeholders’ feeling a sense of ownership toward the SNH and achieving success with priority initiatives.

*One thing that I feel that should impact the care in our institution anywhere is ownership. And ultimately, I am responsible for this patient and I am responsible running this institution or this department, or I may not be the one technically, but if I feel like this is my home or this is my business and this is my institution, I’m gonna do whatever I can to make this work. – Implementation Champion 02*

Nurses, who served in champion and frontline implementation roles, described participation in health disparity-focused initiatives as an appropriate and expected responsibility rather than a burdensome expansion of their job tasks. This synergy between personal motivation and commitment to the mission of a SNH was critical to achieving early buy-in and continued widespread engagement from frontline implementers for proposed initiatives. Participants viewed their commitment to reducing health inequities as a strong driver of change that uniquely exists in the SNH.

*I think that it’s the passion and the motivation behind the work. Like that’s what’s really driving it. And the whole disparity approach… All the people here really love our hospital... They’re here for the shared purpose of like, taking care of our population. So I think that is definitely what is the strongest predictor of success. – Implementation Champion 01*

#### Domain 4: Implementation Processes

The implementation processes of *planning* for and *engaging* stakeholders in priority initiatives were jointly facilitated by multidisciplinary task forces.

#### Facilitator: Multidisciplinary task forces conducted successful implementation efforts

Many initiatives were conceptualized and planned by multidisciplinary committees or steering groups comprised of hospital leadership, providers, and staff at different levels. Multidisciplinary teams were described as “what you need to be effective” (Implementation Champion 12). Bringing diverse stakeholders into the decision-making process enabled teams to see how new initiatives would differentially affect physician and nurse workflows.

*I think it’s a big error when people don’t have multidisciplinary. Even if you don’t think that you need multidisciplinary, I think you don’t really know what they can contribute until you’ve allowed them to contribute. – Frontline Implementer 15*

Multidisciplinary teams also facilitated quick and effective pre-implementation planning since stakeholders were able to collectively brainstorm potential challenges such as differences in discipline-specific readiness for change and design appropriate implementation strategies. Multidisciplinary teams also provided an opportunity for stakeholders to discuss the evidence for an innovation from different perspectives, providing implementation champions with multiple approaches to promote widespread buy-in.

*We had a lot of different players at the table that really made it more efficient than trying to say, ‘oh we gotta do this’. And then after the fact going, ‘oh we can't do this’, ‘we can’t do that’, by different players. The players were at the table. So that, to me, made a huge difference than previous experiences with trying to develop projects. – Implementation Champion 19*

## Discussion

This research aimed to identify barriers and facilitators to implementing priority initiatives in a SNH where organizational culture, resources, patient needs, and other implementation factors may differ from non-SNHs. Although key informants described diverse interventions to improve healthcare delivery, their experiences converged on seven themes including three barriers and four facilitators related to four CFIR domains: inner setting, outer setting, characteristics of individuals, and implementation process.

### Barriers

The three barriers identified in our analysis negatively impacted initiatives throughout all stages of implementation by delaying initial uptake, requiring unplanned implementation strategy adaptations, and hindering sustainability.

Limitations on available resources to staff initiatives or to financially invest in sustaining successful efforts were identified as barriers to every priority initiative identified in our analysis. These inner setting barriers are consistent with prior research documenting the impact of time and financial constraints on delayed implementation progress [31–33] in SNHs and non-SNHs. In our study, implementation champions and frontline implementers reported a lack of protected time to plan implementation efforts and the inability to hire analytic support to monitor progress. These barriers appear to have a bigger impact on implementation champions and frontline implementers carrying out initiative work than on implementation overseers whose involvement is more distal.

Research by Bickell et al. suggests that some providers may identify creative workarounds to navigate time and other resource deficiencies, but this forced flexibility may also promote burnout [34]. Limited time and resources may serve as a greater barrier to implementation efforts in safety net settings that may be understaffed and require providers to absorb multiple clinical and administrative roles [31, 35, 36] than in non-SNHs.

We also found a tension between stakeholders’ appreciation of congratulatory organizational recognition for their improvement efforts and feeling frustrated over the lack of financial reinvestment in successful initiatives. This barrier was most reported by implementation overseers who may be directly involved in negotiating department budgets with the SNH leadership.

SNHs may have fewer resources to invest in initiatives and may be simultaneously more risk averse to investing in change. Prior research in public, non-profit, and under-resourced agencies have found such implementation climates to generally have less favorable attitudes toward change [37, 38] and leadership with lower risk tolerance [39] compared to private, for-profit agencies. Risk aversion may prevent SNHs from adopting evidence-based practices including innovations that could result in greater long-term savings.

Research indicates that higher rates of perceived organizational support, such as the laudatory emails identified in our study, are associated with increased employee empowerment, job satisfaction, and performance [40, 41]. However, our study results suggest that congratulatory recognition and organizational resources (e.g., financial investment, staff, protected time) are necessary to sustain improvement gains. SNHs might consider allocating limited funding based on internal metrics that evaluate the success of piloted improvement efforts and their overall alignment with organizational goals. Such arrangements would likely require negotiations between implementation overseers and SNH leadership. Implementation overseers could also use departmental resources to establish creative team structures that partner initiative implementation champions with team members skilled in project management, data collection, and analysis.

Although stakeholders had a high degree of knowledge about SNH patients’ complexity, implementation strategies that did not adequately navigate patient needs ended up delaying initiative progress. Prior research has examined how outer setting characteristics such as patient needs [42], inter-organizational competitive pressure [43], and external policies [44, 45] influence implementation designs and success. SNHs often serve a greater proportion of lower income, racially diverse patients with complex comorbidities than do non-SNHs [46]. Our findings highlight the need for SNHs to consider how implementation strategies must be compatible with outer setting dynamics like the complexities of the patients they serve, which may extend beyond immediate their healthcare needs.

### Facilitators

Four facilitative themes were identified across four CFIR domains in our study indicating that despite a concentration of inner and outer setting barriers, positive factors abound to promote implementation successes.

In the present study, participants from all stakeholder groups frequently described having a personal creed and commitment to reduce health inequities for their SNH patients. Similarly, a prior national survey found that safety net providers were twice as likely to report looking for racial and ethnic disparities within their practice as compared to non-safety net providers [47]. However, the survey also found no significant difference in providers’ attitudes toward or participation in initiatives addressing vulnerable populations [47]. In contrast, we found that individuals’ personal desire to improve health outcomes for vulnerable populations motivated them to support and actively participate in new initiatives. The prior national survey was limited to primary care providers while our study included hospital physicians, nurses, and leadership in inpatient settings, where the effects of socioeconomic inequities present as immediately life-threatening illness.

The prevalence of healthcare providers reporting a personal commitment to reducing health disparities is likely related to individual’s choice to practice medicine in SNHs. Walker et al. similarly identified personal attributes including shared self-identify with safety net communities and moral obligation as drivers of physicians decision to work with vulnerable populations and in health provider shortage areas [48]. Thus, personal motivation to reduce health inequities may be a stronger driver of improvement initiatives in SNHs than non-SNHs.

Establishing multidisciplinary task forces that engaged diverse stakeholders to plan, execute, and monitor progress toward implementation goals was identified as another facilitator across initiatives. Stakeholders valued multidisciplinary task forces because they invited a variety of perspectives to proactively address potential implementation barriers. Research suggests that teams comprised of diverse expertise and personalities may enhance overall team creativity and performance [49, 50]. Due to the hierarchical structure of most medical teams [51], multidisciplinary task forces may provide greater opportunities for staff and nurses to serve in leadership positions and generate widespread buy-in across disciplines. In both SNH and non-SNHs, implementation overseers may need to play a larger role in ensuring multidisciplinary task forces are established.

Although multidisciplinary task forces can be used in any setting, these groups may be critical to SNHs where lower staffing [31, 35, 36] and a higher volume of patients with complex needs [18, 52] may make it harder to step away from clinical duties to participate in all aspects of implementation. Multidisciplinary task force members can share decision-making and focus on implementation issues that require their expertise, thereby making their involvement valuable and manageable. This may be particularly important in SNHs. In our study, stakeholders’ knowledge of their safety net patients’ complex biopsychosocial needs facilitated the development of and support for new initiatives targeting health inequities, but did not always translate into well-designed implementation strategies. Multidisciplinary teams may promote greater knowledge exchange about patient needs and promote more appropriate implementation strategies to manage those needs.

Finally, effective communication has been widely identified as an influential inner setting dynamic for implementation efforts [33, 34]. Our results reinforce the imperative to identify local communication styles that enable stakeholders to effectively share knowledge and information about implementation activities. The use of passive communication strategies alone resulted in poor stakeholder awareness or understanding of practice changes. Our findings are consistent with a prior meta-analysis that identified didactic education, guideline posting, and printed materials as ineffective methods for information sharing in healthcare settings [53]. Passive communication efforts may fail to spread knowledge if messaging is untargeted and not tailored to stakeholders’ needs. However, passive dissemination has been effective in settings where implementers have an initially high level of expertise and confidence, an effective champion driving change, and adequate professional resources [54]. The ability to share information effectively using passive communications is thwarted unless all three conditions are met [54]. Although all of the initiatives identified in our study had champions, each implementation effort reported insufficient resources—a barrier that may be common to SNHs and negate the benefits of passive-only communications.

We found that stakeholders achieved greater fidelity to practice changes when initiatives combined passive and active communication styles. However, both passive and active approaches have documented drawbacks—passive communication via word of mouth or printed materials may be more affordable but less effective, while active, participatory educational approaches may be more effective, costly, and time-consuming [53]. Our results suggest that SNH conscientious of balancing costs and effective communication should employ a combination of didactic and interactive approaches tailored to the needs of their stakeholders.

### Implications for practice

SNHs and non-SNHs may experience similar resource constraints that delay or fail to sustain improvement initiatives. However, the financial constraints may be enhanced for SNHs operating with marginal profits. Future SNH efforts should focus on enhancing inner setting dynamics including hiring staff with research design, data collection, analysis, and project management capacity to mitigate these challenges. Additionally, SNHs may benefit from drivers of change that are unique to the safety net such as provider’s personal motivation and competitiveness to reduce health disparities. SNHs should promote the use of multidisciplinary task forces to achieve a holistic understanding of SNH barriers, leverage diverse skills across team members, and promote effective communication strategies to achieve implementation goals.

### Strengths and limitations

Our study demonstrates the feasibility of using CFIR to identify common barriers and facilitators across multiple initiatives and inner setting dynamics, while harnessing familiar implementation science terminology to promote greater transferability of findings. A prior systematic review of CFIR applications in implementation science research suggested that CFIR’s standardized language might be helpful for comparing varied initiatives; however, this hypothesis lacked evidence [24]. Our study provides the necessary evidence for a macro-application of CFIR. Additional study strengths include having a diverse sample of stakeholders in multiple SNH positions and implementation roles. Double-coding transcripts provided a rigorous, consistent application of CFIR codes. However, using a directed content approach rather than inductive coding may have restricted us from identifying non-CFIR-related themes important to implementation. The breadth and inclusivity of CFIR constructs may negate this limitation. Interviewees’ recall bias may limit this research since initiatives began months before our interview. This research represents the experiences of one urban, academic SNH. Themes identified here may not be transferable to other SNHs operating in rural settings or without medical school affiliation.

## Conclusion

This study identified three barriers to adopting and sustaining new initiatives in a SNH: [1] limited staffing resources, [2] organizational recognition without financial investment, and [3] use of implementation strategies that did not adequately address patients’ biopsychosocial complexities. Four facilitating themes were also identified [1] implementation approaches that combined passive and active communication styles, [2] knowledge of patient needs and competitive pressure to perform well against non-SNHs, [3] stakeholders’ personal commitment to reduce health inequities, and [4] the use of multidisciplinary task forces to drive implementation activities. Additionally, we demonstrated the applicability of CFIR at a macro-level to identify common themes across diverse initiatives and settings.

## Supplementary information

**Additional file 1: Supplemental Appendix 1.** Barriers and Facilitators to Implementing Priority Inpatient Initiatives in the Safety Net Setting: Interview Guide. This document contains the full qualitative interview guide used to collect data reported in this manuscript.

**Additional file 2: Supplemental Appendix 2.** Standards for Reporting Qualitative Research Checklist. This document contains the completed Standards for Reporting Qualitative Research checklist for the reported study.

## Data Availability

The qualitative data generated and/or analyzed during the current study are not publicly available because they were generated in interviews with the research team, with the expectation that participant identity would be kept confidential. De-identified transcripts may be available from the corresponding author on reasonable request.

## Abbreviations

CFIR: Consolidated Framework for Implementation Research
DSH: Disproportionate Share Hospital
SNH: Safety net hospital
