# Supplementary material for: Barriers and facilitators to implementing priority inpatient initiatives in the safety net setting
Source: Implement Sci Commun. 2020 Mar 11;1:35. doi: 10.1186/s43058-020-00024-6 (PMC7427845; doi:10.1186/s43058-020-00024-6)
Supplement: Supplementary file 1 — Additional file 1: Supplemental Appendix 1. Barriers and Facilitators to Implementing Priority Inpatient Initiatives in the Safety Net Setting: Interview Guide. This document contains the full qualitative interview guide used to collect data reported in this manuscript. [file 43058_2020_24_MOESM1_ESM.docx]

**Barriers and Facilitators to Implementing Priority Inpatient Initiatives in the Safety Net Setting: Interview Guide**

## Warm-Up Questions:

1. [Discuss goals of this research]. Do you have any additional questions about this project before we get started?
2. What are the major responsibilities in your current position?

##

## Discussion Questions

*Probes are included as bullets for each primary question.*

1. Describe the most important initiative that the Section tried to implement in the last 12 months.
2. Describe how the initiative was developed.
   - Who was involved?
   - What evidence was used to predict successful implementation in your Section?
   - How was the initiative communicated?
   - Describe any feedback/review process.
3. Why was this initiative selected or prioritized?

- How much of a priority is this initiative for you compared to Section leadership? Why?
- Who deemed it a priority?

1. What population is the best focus for this initiative? Why?

- Target patient population? All patients?
- Certain provider types?

1. Describe how the initiative was implemented.
   - What strategies were used (e.g. communications, trainings, incentives, champions)?
   - Were any implementation goals set?
   - How receptive was the Section to implementation?
   - Describe any feedback/review process.
2. Describe your role in the initiative and its implementation.

- What is your level of engagement?
- How do you feel about it being implementing in your setting?

1. Describe any feedback processes used to evaluate implementation strategies.
   - What measures were tracked (e.g. patient outcomes, process, clinician/staff response)?
   - How did you provide/receive feedback?
   - What kind of changes were made based on feedback?
2. What do you think is working regarding the implementation of this initiative? Why?
   - Facilitators for feasibility (e.g. simplicity of strategies, resources, cost, learning environment)?
   - Key strategies/components that should be preserved?
3. What do you think is not working regarding the implementation of this initiative? Why?
   - Feasibility barriers (e.g. complexity of strategies, resources, cost, learning environment)?
   - Key strategies/components that should be altered?
4. Describe any milestone successes related to implementing the initiative.
   - Why/how did you achieve these successes?
5. Describe any major challenges and facilitators for implementation efforts as they relate to:
   - The safety net hospital
   - The section/inpatient setting where you work
   - Personnel/colleagues (e.g. change fatigue/burnout, staffing resources)
   - Patient population
   - External policies/incentives (e.g. Medicaid policy, performance measures, guidelines)
   - Other
6. What recommendations can you suggest to address the aforementioned challenges?
7. If you could change anything at this hospital in order to make it easier for others to accomplish a similar initiative, what would you change?

- To similar initiatives?
- To other initiatives?

1. How do the implementation strategies used for this priority initiative compare to strategies used in prior initiatives?
   - Advantages/disadvantages compared to past efforts?
2. What have you learned from trying to implement this initiative?

- Sustainability
- Suggestions for others implementing similar initiatives in inpatient settings
  - At this safety net hospital
  - At other healthcare facilities

1. Is there anything else you would like to discuss that has not been addressed?
